# Gallic Acid Potentiates the Antimicrobial Activity of Tulathromycin Against Two Key Bovine Respiratory Disease (BRD) Causing-Pathogens

**DOI:** 10.3389/fphar.2018.01486

**Published:** 2019-01-04

**Authors:** Karthic Rajamanickam, Jian Yang, Meena Kishore Sakharkar

**Affiliations:** Drug Discovery and Development Research Group, College of Pharmacy and Nutrition, University of Saskatchewan, Saskatoon, SK, Canada

**Keywords:** bovine respiratory disease, gallic acid, tulathromycin, dairy and beef cattle, antimicrobial resistance

## Abstract

Bovine respiratory disease (BRD) is the most common infectious disease in dairy and beef cattle. It is associated with significant morbidity and mortality and causes a huge economic loss each year. In western Canada, a one-time injection of tulathromycin is commonly used as a metaphylactic procedure to reduce BRD incidence and eliminate potential BRD outbreak. With increased global concern on antimicrobial usage in dairy and beef products and bacterial resistance to antimicrobials, it is important to develop a novel strategy to eliminate the usage or decrease the dosage of antimicrobials. In this study, we showed that gallic acid was active against both *Mannheimia haemolytica* and *Pasteurella multocida*, two key BRD associated-pathogens, with the minimum inhibitory concentration (MIC) measured at 250 and 500 μg/mL, respectively. Co-administration of tulathromycin and gallic acid exhibited a strong additive or weak synergistic effect toward both *M. haemolytic* and *P. multocida*. Tulathromycin, gallic acid and their combination were also effective against the mixed culture of *M. haemolytic* and *P. multocida*. Furthermore, we showed that pre-exposure to tulathromycin generated bacterial resistance to the antimicrobial in *M. haemolytica* but not in *P. multocida*.

## Introduction

Bovine respiratory disease (BRD) is the most prevalent infectious disease in diary and beef cattle (Griffin, 1997). BRD imposes deleterious effects on cattle health and performance, resulting in substantial economic loss (Wittum et al., 1996; Smith, 1998; Larson, 2005; Rajala-Schultz et al., 2009; Johnson and Pendell, 2017). For example, the annual loss caused by BRD is about $US 600–750 million to the North American beef industry. BRD is commonly regarded as a multi-factorial disease, with bacterial infection, viral infection, and stress as the three major co-dependent factors (Holman et al., 2017; Headley et al., 2018). The key pathogenic bacteria identified in Canada are *Mannheimia haemolytica, Pasteurella multocida* and *Haemophilus somni* (Ellis, 2001; Apley, 2006). Vaccines have been developed against these three pathogens; however, the vaccination result is not consistent (Larson and Step, 2012). In western Canada, new feedlot placements usually get a metaphylactic injection of tulathromycin (Draxxin) to reduce the risk and severity of BRD upon arrival (Lindsay, 2003; Checkley et al., 2010). Tulathromycin is a semi-synthetic triamilide antimicrobial, which inhibits the synthesis of essential bacterial proteins (Schunicht et al., 2007).

The practice of metaphylactic injection of antimicrobials may lead to the development of antimicrobial resistance (AMR), which, in turn, will reduce the efficacy of the antimicrobials commonly employed to control infectious disease, such as BRD, in cattle (Checkley et al., 2010). Here, it is important to mention that subinhibitory concentrations of antimicrobials have been suggested to be associated with generation of genotypic and phenotypic variability and has been used for selection of bacteria resistant to antimicrobials (Andersson and Hughes, 2014). Antimicrobials at subinhibitory concentrations have been reported to function as signaling molecules causing alterations in bacterial physiology e.g., alterations in breakpoint, virulence, biofilm formation, gene transfer, etc (Bhattacharya et al., 2017). Furthermore, with increased concern from the consumers about antimicrobial usage in dairy and beef products, Health Canada has decided to introduce a new regulation that a veterinary prescription is required to purchase any livestock antimicrobial from December (West-Central Forage Association, 2018). Thus, there is an urgent need to identify alternatives of antimicrobials, such as natural products, and develop novel and effective treatment protocols with significantly reduced usage, and dosage of antimicrobials in order to minimize the development of AMR. For example, controlling pulmonary inflammation with a non-steroidal anti-inflammatory drug (NSAID) is critical to avoid irreversible lung damage in the cattle other than the antimicrobial treatment of BRD (Lindsay, 2003).

Plants synthesize a diverse array of secondary metabolites (phytochemicals), which are not only involved in self-defense but also possess a wide range of health-promoting effects such as antimicrobial activities. The use of antimicrobials in combination with phytochemicals has been extensively studied (Monden et al., 2002; Petersen et al., 2006; Černohorská and Votava, 2008). The antimicrobial-phytochemical cocktail strategy has exhibited the potential in eradicating complex pathogens (Neu, 1991; Batista et al., 1994; Cowan, 1999; Lewis and Ausubel, 2006; Wise, 2006; Amyes et al., 2007; Cai et al., 2007; Gould, 2008). Furthermore, majority of phytochemicals are relatively safe for use as compared to purely synthetic drugs due to their natural origins and can be metabolized easily without leaving harmful residues in dairy and beef products. Co-administration of phytochemicals has been shown to enhance antimicrobial activities of antimicrobials and delay the development of AMR. In this study, we evaluated whether gallic acid, a phenolic acid identified in various plants such as gallnuts, possesses antimicrobial activity, potentiates the antimicrobial function of tulathromycin, and reduces or delays AMR to tulathromycin against two key causing-pathogens of BRD, *M. haemolytica* and *P. multocida.*

## Materials and Methods

### Materials

Bacterial strains *M. haemolytica* ATCC 29702 and *P. multocida* ATCC 43137, as well their culture medium (Brain-Heart Infusion broth, BHIB), were purchased from Cedarlane Canada (Burlington, ON, Canada). Gallic acid was purchased from ThermoFisher Scientific (Ottawa, ON, Canada). Tulathromycin A was purchased from Cayman Chemical Company (Ann Arbor, MI, United States). All other chemicals used in this study were purchased from Sigma-Aldrich Canada (Oakville, ON, Canada).

### Determination of Minimum Inhibitory Concentration (MIC)

All experiments in this study were carried out in triplicate. The MICs of tulathromycin and gallic acid against *M. haemolytica* and *P. multocida* were determined using standard broth micro-dilution assay as outlined by the Clinical & Laboratory Standards Institute (CLSI). Both strains were sub-cultured in BHIB at 37°C overnight and then OD_565_ of the bacterial suspensions was adjusted to 0.5 McFarland turbidity with the culture media (approximate cell density: 1.5 × 10^8^ CFU/mL) using normal saline as a control. For each bacterial strain, 100 μL BHIB broth was added to each well of a 96-well plate with subsequent addition of 5 μL/well of the adjusted bacterial suspension. Then, the bacterial samples were treated with either tulathromycin with concentration ranging from 0.04 to 5 μg/mL or gallic acid with concentration ranging from 3.9 to 500 μg/mL. Untreated bacterial samples were used as a negative control. The culture plate was incubated at 37°C for 18–24 h before OD_655_ was taken for each well using a Bio-Rad iMark Microplate Reader (Bio-Rad Laboratories, Inc., Mississauga, ON, Canada). The readings were also double-checked using a Sensititre Vizion Digital MIC Viewing System (ThermoFisher Scientific, Ottawa, ON, Canada).

### Antimicrobial Effect of the Combination of Tulathromycin and Gallic Acid

For each bacterial strain, the antimicrobial activity of the combination of tulathromycin and gallic acid was measured a protocol modified from the one described above. Briefly, the bacterial sample preparation in the 96-well plate was the same. Then, tulathromycin and gallic acid were added to the bacterial sample following a checkerboard serial dilution design. The tulathromycin concentration was 0.08 and 0.16 μg/mL, respectively; and the gallic acid concentration was from 3.91 to 250 μg/mL. The fractional inhibitory concentration (FIC) index of the combination of tulathromycin and gallic acid was calculated according to the *equation 1*. In this equation, FIC_A_ and FIC_B_ are the FIC indices of compounds A and B in the combination, A and B are the MICs of compounds A and B in combination, and MIC_A_ and MIC_B_ are the individual MICs of compounds A and B, respectively. The FIC index determined by checkerboard method is interpreted as following: FIC ≤ 0.5 – synergy; 0.5 < FIC ≤ 4.0 – additivity; and FIC > 4.0 – antagonism.

(1) $$\mathrm{FIC}\;\mathrm{index}={\mathrm{FIC}}_{A}+{\mathrm{FIC}}_{B}=A/MIC_{A}+B/{\mathrm{MIC}}_{B}$$

### Evaluation of Bacterial Resistance Generated From Pre-exposure

In order to evaluate whether bacterial resistance can be generated from pre-exposure to tulathromycin, *M. haemolytica*, and *P. multocida* were cultured in BHIB in the presence of tulathromycin at a dose of 1/2MIC (i.e., 0.16 μg/mL) until OD_565_ reached 1 OD unit of turbidity. Then, the bacterial cells were given a stress relaxation by culturing in BHIB without tulathromycin until OD_565_ reached 1 OD unit of turbidity. The bacterial cells were collected and designated as the 1st generation of pre-exposed cells (1G). The 1G bacterial cells were subjected to the same type of treatment to generate the 2nd generation of pre-exposed cells (2G); and subsequently the 2G bacterial cells were subjected to the same protocol to generate the 3rd generation of pre-exposed cells (3G). In total, we generated three generations (1G, 2G, and 3G) of *M. haemolytica* and *P. multocida* cells pre-exposed to tulathromycin. The MICs of tulathromycin and gallic acid against the 1G, 2G, and 3G *M. haemolytica* and *P. multocida* cells were also measured using the protocol describe above.

### Determination of MIC Against Mixed Culture of *M. haemolytica* and *P. multocida*

The MICs of tulathromycin and gallic acid against the mixed culture of *M. haemolytica* and *P. multocida* were determined using the same protocol described above except that both strains were mixed cultured in BHIB at 37°C overnight and OD_565_ of the mixed cultured bacterial suspension was adjusted to 0.5 McFarland turbidity with the culture media. The antimicrobial effect of the combination of tulathromycin and gallic acid against the mixed culture of *M. haemolytica* and *P. multocida* was also measured using the checkerboard serial dilution method. The tulathromycin concentration was 0.16, 0.31, and 0.62 μg/mL, respectively; and the gallic acid concentration was from 3.91 to 500 μg/mL.

### Statistical Analysis

All the experiments were performed in triplicate. Data of experimental results was recorded as mean ± standard deviation. One way ANOVA *t*-test was used for statistical significance and a *P*-value < 0.05 (denoted as ^∗^) was regarded as significant. Correlation coefficient (*r*) value calculated by using Pearson’s Correlation method. Statistical analyses were performed using Graph Pad Prism 5.0 statistical software.

## Results

### MICs of Tulathromycin and Gallic Acid

**FIGURE 1:**
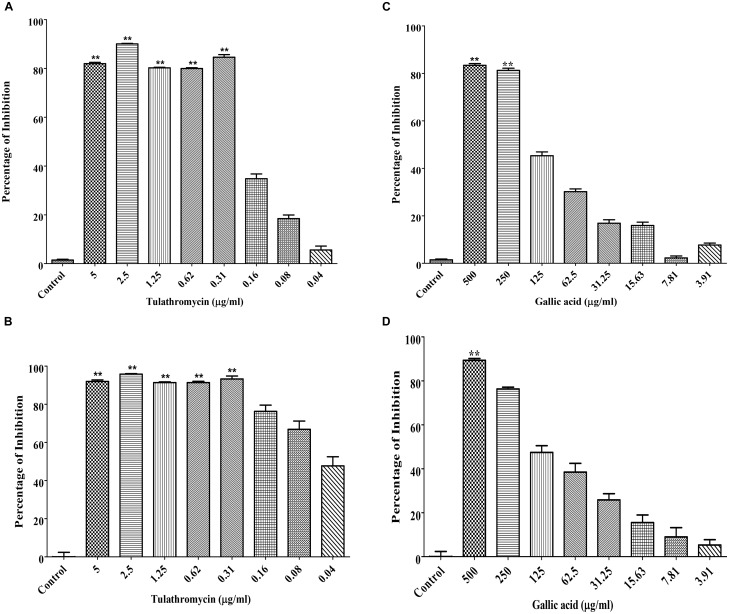
Determination of the MICs of tulathromycin against *M. haemolytica* **(A)** and *P. multocida* **(B)** and the MICs of gallic acid against *M. haemolytica* **(C)** and *P. multocida* **(D)**. The concentration of tulathromycin was ranging from 0.04 to 5 μg/mL and the concentration of gallic acid was ranging from 3.9 to 500 μg/mL. Bacterial cell culture medium, Brain-Heart Infusion broth (BHIB), was used as a negative control. One way ANOVA *t*-test was used for statistical significance. ^∗^*P*-value < 0.05, ^∗∗^*P*-value < 0.01, and ^∗∗∗^*P*-value < 0.001 are shown. Correlation coefficient (*r*) was calculated using Pearson’s correlation method.

The MIC of tulathromycin was determined to be 0.31 μg/mL against both *M. haemolytica* and *P. multocida* (Figures 1A,B). The MIC of gallic acid was determined to be 250 μg/mL against *M. haemolytica* (Figure 1C) and 500 μg/mL against *P. multocida* (Figure 1D).

### Strong Additive or Weak Synergistic Effect Between Tulathromycin and Gallic Acid

**FIGURE 2:**
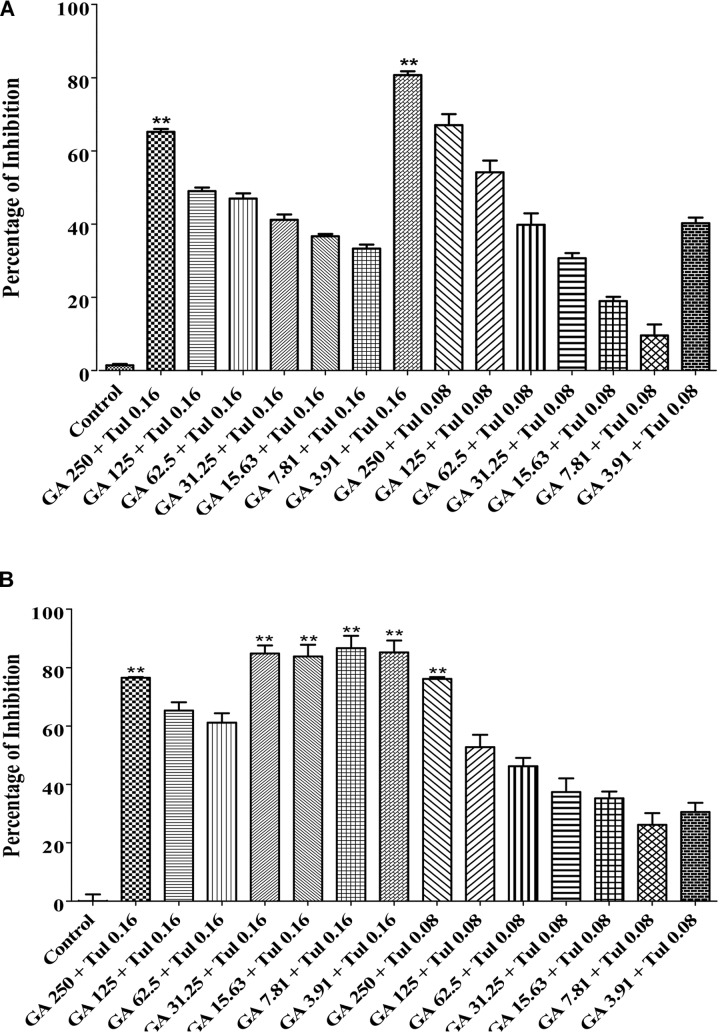
Antimicrobial activity of the combination of tulathromycin and gallic acid against *M. haemolytica* **(A)** and *P. multocida* **(B)**. The concentration of tulathromycin was 0.08 and 0.16 μg/mL, respectively, and the concentration of gallic acid was from 3.91 to 250 μg/mL. Bacterial cell culture medium, BHIB, was used as a negative control. One way ANOVA *t*-test was used for statistical significance. ^∗^*P*-value < 0.05, ^∗∗^*P*-value < 0.01, and ^∗∗∗^*P*-value < 0.001 are shown. Correlation coefficient (*r*) was calculated using Pearson’s correlation method.

The checkerboard broth micro-dilution assay was carried out to investigate whether co-administration of tulathromycin and gallic acid could exhibit synergistic/additive effect. The antimicrobial activity against *M. haemolytica* and *P. multocida* was evaluated for tulathromycin at two sub-MIC doses (0.16 and 0.08 μg/mL), gallic acid at seven sub-MIC doses (250, 125, 62.5, 31.25, 15.6, 7.8, and 3.9 μg/mL), and their combination. For *M. haemolytica*, tulathromycin gave a 37% inhibition of growth at concentration of 0.16 μg/mL and gallic acid showed an 8% inhibition of growth at concentration of 3.91 g/mL (Figure 2A). However, co-administration of 0.16 μg/mL tulathromycin and 3.91 μg/mL gallic acid dramatically increased the inhibition of growth to 81% (Figure 2A). The FIC index of the combination of tulathromycin and gallic acid was 0.5, implicating a strong additive or weak synergistic effect between tulathromycin and gallic acid agsint *M. haemolytica*. For *P. multocida*, tulathromycin exhibited a 77% inhibition of growth at concentration of 0.16 μg/mL and gallic acid gave a 5% inhibition of growth at concentration of 3.91 μg/mL (Figure 2B). Co-administration of 0.16 μg/mL tulathromycin and 3.91 μg/mL gallic acid enhanced the inhibition of growth to 91% (Figure 2B). The FIC index of the combination of tulathromycin and gallic acid was calculated to be 0.5, implicating that there is also a strong additive or weak synergistic effect between tulathromycin and gallic acid agsint *P. multocida*. The mixed culture of *M. haemolytica* and *P. multocida*, tulathromycin gave a 70.6% inhibition of growth at concentration of 0.16 μg/mL (Figure 5A). However, gallic acid showed 38.5% inhibition of growth at a concentration of 31.25 μg/mL (Figure 5B). Co-administration of 0.16 μg/mL tulathromycin and 31.25 μg/mL gallic acid reasonably increased the inhibition of growth to 74.2% (Figure 5C). The FIC index of the combination of tulathromycin and gallic acid was 0.56 suggesting a strong additive or weak synergistic effect between tulathromycin and gallic acid against the mixed culture of *M. haemolytica* and *P. multocida*. Other combinations showed indifferent effect.

### Bacterial Resistance Generated From Pre-exposure

**FIGURE 3:**
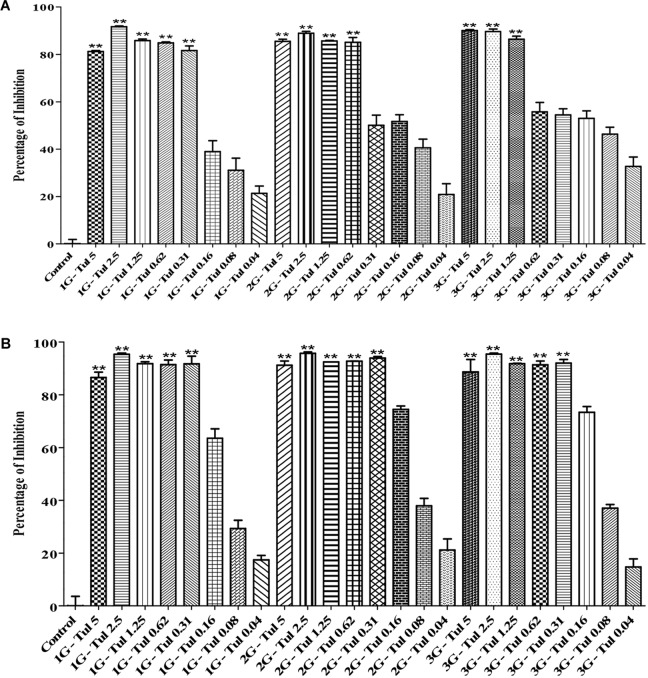
Determination of the MICs of tulathromycin against the 1G, 2G and 3G cells of *M. haemolytica* **(A)** and the 1G, 2G, and 3G cells of *P. multocida* **(B)**, which had been pre-exposed to tulathromycin at 1/2MIC concentration for one, two and three times, respectively. The concentration of tulathromycin was ranging from 0.04 to 5 μg/mL. One way ANOVA *t*-test was used for statistical significance. ^∗^*P*-value < 0.05, ^∗∗^*P*-value < 0.01, and ^∗∗∗^*P*-value < 0.001 are shown. Correlation coefficient (*r*) was calculated using Pearson’s correlation method.

**FIGURE 4:**
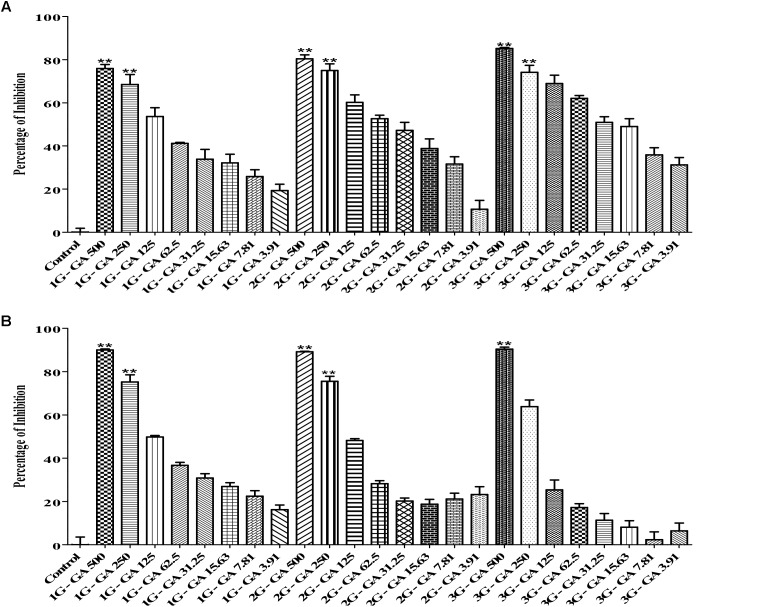
Determination of the MICs of gallic acid against the 1G, 2G, and 3G cell of *M. haemolytica* **(A)** and the 1G, 2G and 3G cells of *P. multocida* **(B)**, which had been pre-exposed to tulathromycin at 1/2MIC concentration for one, two, and three times, respectively. The concentration of gallic acid was ranging from 3.9 to 500 μg/mL. One way ANOVA *t*-test was used for statistical significance. ^∗^*P*-value < 0.05, ^∗∗^*P-*value < 0.01, and ^∗∗∗^*P*-value < 0.001 are shown. Correlation coefficient (*r*) was calculated using Pearson’s correlation method.

In this study, we generated three generations (1G, 2G, and 3G) of *M. haemolytica* and *P. multocida* samples that had been exposed to either tulathromycin or gallic acid for one, two and three times, respectively. As shown in Figure 3, the MIC of tulathromycin was measured to be 0.31 μg/mL for 1G, 0.62 μg/mL for 2G, and 1.25 μg/mL for 3G, respectively, for *M. haemolytica* and 0.31 g/mL for all three generations of pre-exposed *P. multocida*. This implicated that pre-exposure to tulathromycin at 1/2MIC (0.16 μg/mL) induced bacterial resistance in *M. haemolytica* but not in *P. multocida*. Furthermore, the bacterial resistance was positively correlated with the pre-exposure times of tulathromycin for *M. haemolytica*. For gallic acid, the MIC was measured to be 250 μg/mL for all three generations of pre-exposed *M. haemolytica* and 250 μg/mL for 1G, 250 μg/mL for 2G, and 500 μg/mL for 3G, respectively, for *P. multocida* (Figure 4). Therefore, pre-exposure to tulathromycin did not alter the sensitivity of *M. haemolytica* but sensitized *P. multocida* toward gallic acid. Further studies are warranted to confirm whether this sensitization toward gallic acid is true and how it has happened in *P. multocida*.

Absorbance data is available upon request.

## Discussion

Bovine respiratory disease (BRD), which is associated with morbidity and mortality, is the most common infectious disease in cattle and a significant threat to dairy and beef industry. Mass antimicrobial treatment upon animal arrival is widely used to reduce incidence of BRD and eliminate/minimize a potential outbreak (Taylor et al., 2010). However, the increased global concern on antimicrobial abuse in the dairy and beef industry has led many countries to ban or limit antimicrobials as growth promoters (McVey, 2009; Urban-Chmiel and Grooms, 2012; Johnston et al., 2017). In western Canada, tulathromycin is normally used as the metaphylactic agent to reduce the risk and severity of BRD. Resistance to tulathromycin, conferred by rRNA mutations, has been found in field isolates of *M. haemolytica* and *P. multocida* (Wadood et al., 2017; Uddin et al., 2018). Therefore, it is important to develop a novel strategy to decrease the dosage of tulathromycin, which, in turn, would reduce or eliminate the generation of resistance by the bacterial pathogens. One effective way to reduce antimicrobial dosage and delay AMR is to use a cocktail of two antimicrobials or a cocktail of an antimicrobial and a phytochemical.

Phytochemicals, which are antimetabolites produced in plants, have provided a valuable resource for relatively cheap and safe antimicrobial agents. The toxicity of phytochemicals is normally low and very few adverse effects have been reported (Nascimento et al., 2000). It has been shown that gallic acid, a phenolic acid, not only possesses antimicrobial activity against various bacteria such as *Pseudomonas* strains but also potentiates the efficacy of antimicrobials (Al-Abd et al., 2015; Fu et al., 2016; Samad et al., 2016). Previous studies have also demonstrated that gallic acid inhibited efflux pumps, which are a major mechanism in generating AMR, in *Staphylococcus aureus* resistant strains and multidrug resistant *Escherichia coli* strains (Simoes et al., 2009; Abreu et al., 2012). In addition, epigallocatechin gallate, which is an ester of epigallocatechin and gallic acid, has been shown to exhibit antifolate activity against *Stenotrophomonas maltophilia* (Navarro-Martínez et al., 2005). Therefore, the strong additive or weak synergistic effect between tulathromycin and gallic acid against *M. haemolytica* and *P. multocida* is likely through the inhibitory effect of gallic acid on efflux pumps and antifolate activity. Further studies are warranted to confirm whether gallic acid can indeed inhibit efflux pumps and folate synthesis in *M. haemolytica* and *P. multocida*. It is also noteworthy that gallic acid is an arginase inhibitor and arginase has been shown to be regulated in allergic lung disease (Pudlo et al., 2017). Since controlling pulmonary inflammation with an NSAID is critical to avoid irreversible lung damage in the cattle infected with BRD (Pal et al., 2010), the anti-inflammatory activity of gallic acid *via* inhibiting arginase would provide an extra benefit in treating BRD.

**FIGURE 5:**
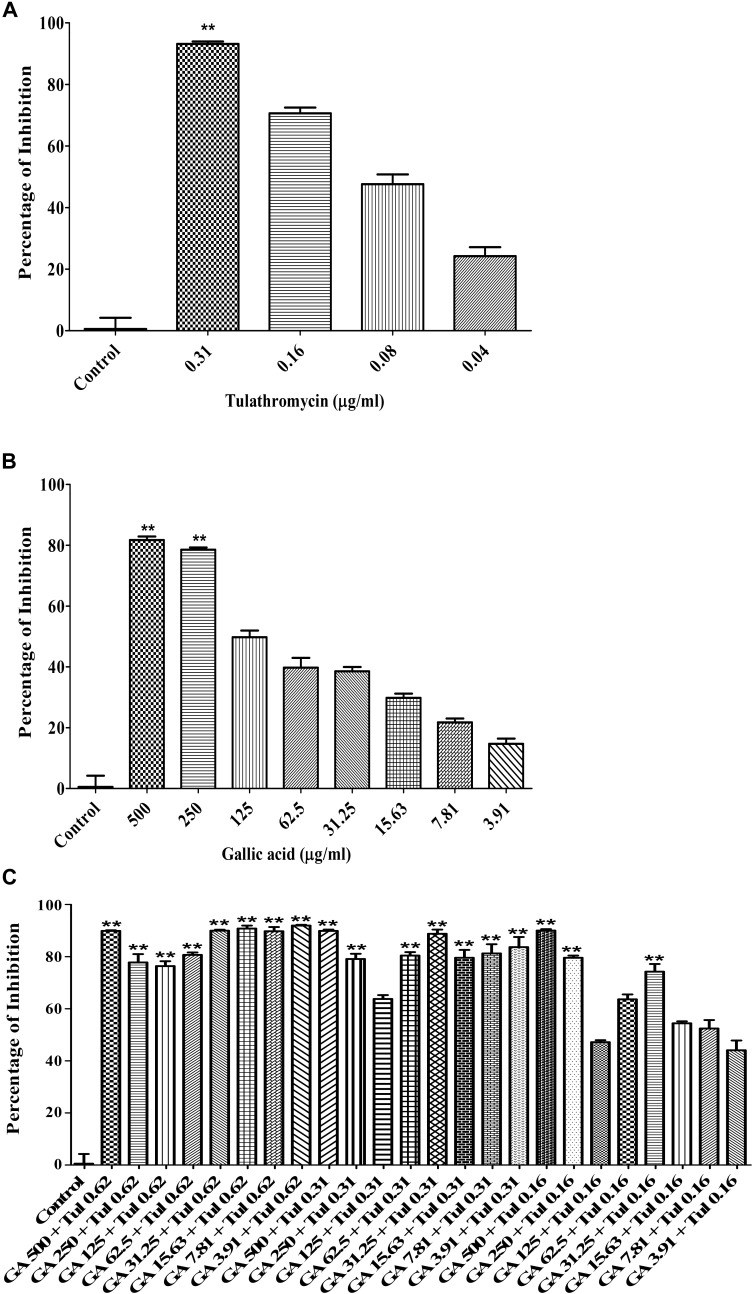
Antimicrobial activity of tylathromycin [**(A)** concentration: 0.04 – 0.31 μg/mL], gallic acid [**(B)** concentration: 3.91 – 500 μg/mL] and combination of tulathromycin and gallic acid [**(C)** concentration: tulathromycin 0.16, 0.31, and 0.62 μg/mL and gallic acid 3.91 – 500 μg/mL] against the mixed culture of *M. haemolytica* and *P. multocida*. One way ANOVA *t*-test was used for statistical significance. ^∗^*P*-value < 0.05, ^∗∗^*P*-value < 0.01, and ^∗∗∗^*P*-value < 0.001 are shown. Correlation coefficient (*r*) was calculated using Pearson’s correlation method.

Because BRD is a multi-factorial syndrome and more than one bacteria may be involved in the disease simultaneously, we decided to evaluate whether tulathromycin, gallic acid, and/or their combination were effective against the mixed culture of *M. haemolytica* and *P. multocida*. The MIC of tulathromycin was found to be 0.31 μg/mL against the mixed culture (Figure 5A), which is the same as that against each individual bacterium. The MIC of gallic acid was found to be 500 μg/mL against the mixed culture (Figure 5B), which is the same as that against *P. multocida* but higher than that against *M. haemolytica*. Co-administration of 0.16 μg/mL tulathromycin and 500 μg/mL gallic acid produced the maximum inhibition of the mixed culture (Figure 5C). Therefore, we concluded that tulathromycin, gallic acid and their combination are also effective against the mixed culture of *M. haemolytica* and *P. multocida*. Further validation of the current data with clinical isolates of *M. haemolytica* and *P. multocida* and investigation with an *in vivo* animal model may help in determining whether the co-administration approach with gallic acid is indeed clinically practical in reducing dosage of tulathromycin and delay/prevent the emergence of AMR in feedlots.

## Conclusion

Bovine respiratory disease (BRD) is the most common infectious disease in dairy and beef cattle. In this study, we showed that both tulathromycin and gallic acid were effective against *M. haemolytica, P. multocida*, and their mixed culture. A strong additive or weak synergistic effect was observed between tulathromycin and gallic acid against both bacteria. In addition, pre-exposure to tulathromycin generated bacterial resistance to the antimicrobial in *M. haemolytica* but not in *P. multocida*.

## Author Contributions

This work was designed by MS and JY carried out by KR. The manuscript was prepared by MS and JY and approved by all authors.

## Conflict of Interest Statement

The authors declare that the research was conducted in the absence of any commercial or financial relationships that could be construed as a potential conflict of interest.
